# Feasibility of using continuous positive airway pressure *via* the LeVe CPAP System among children with acute hypoxaemic respiratory failure at Mengo Hospital, Kampala, Uganda: a mixed-methods study

**DOI:** 10.1183/23120541.00673-2024

**Published:** 2025-06-30

**Authors:** Edith Namulema, William Davis Birch, Rhoda Nakiriba Mayega, Barbara Namugga, Racheal Musasizi, Ambrose Tumwesigye, Anna Littlejohns, Helen Please, Vishal Sharma, Alice Cunningham, Ian Waters, David Brettle, Jiten D. Parmar, Roy Miller, Tim Beacon, Stuart Murdoch, Peter Culmer, Nikil Kapur, Mark Winton, Tom Lawton

**Affiliations:** 1 Mengo Hospital, Kampala, Uganda; 2 University of Leeds, School of Mechanical Engineering, Leeds, UK; 3 Leeds Teaching Hospitals NHS Trust, Leeds, UK; 4 Bradford Royal Infirmary, Bradford, UK; 5 Medical Aid International, Stagsden, UK

## Abstract

**Background:**

Continuous positive airway pressure (CPAP) is a well-established treatment modality for children in moderate and severe respiratory failure in well-resourced settings. However, the availability of CPAP is generally poor in many resource-limited settings, in large part because existing CPAP devices are not designed for cost and resource efficiency, which precludes their use. The LeVe CPAP System has been co-developed by an international multidisciplinary team specifically for use in low-resource settings. In this paper we report the first study evaluating the efficacy of using the LeVe CPAP System as an intervention for children with acute hypoxaemic respiratory failure at Mengo Hospital in Kampala, Uganda.

**Methods:**

A total of 42 paediatric patients were recruited onto the study, all of whom were failing to maintain oxygen saturation above 88% at room conditions. Key clinical measures, including oxygen saturation, heart rate, respiratory rate and dyspnoea were recorded every hour for the length of admission on the paediatric ward.

**Results:**

At completion, 39 patients had recovered and were successfully discharged while 3 of 42 (7%) died in the early phases of treatment. Surviving patients showed improvements in all clinical measures, particularly in the first 12 h of treatment, and no adverse effects were reported after continued use. Additionally, we interviewed five parents whose children were undergoing treatment to gain a qualitative assessment of perceptions to the LeVe CPAP System.

**Conclusion:**

Outcomes of the study demonstrate the capability of the LeVe CPAP System to treat paediatric patients in respiratory failure and support the system's wider adoption in low-resource settings.

## Shareable abstract

**The LeVe CPAP System provides effective respiratory support for paediatric patients with hypoxaemic respiratory failure in low-resource contexts, improving patient outcomes and minimising the need for further treatment escalation** https://bit.ly/40kPZBU

## Introduction

Acute hypoxaemic respiratory failure (AHRF) is defined as severe hypoxaemia without hypercapnia [1] and is among the most common causes of critical illness with a high hospital mortality [2]. While AHRF can be due to several underlying conditions, pneumonia accounts for over 60% of cases [1]. Pneumonia is the greatest cause of death in children younger than 5 years globally, accounting for 12.8% of all deaths beyond the neonatal period [3]. There is an increased prevalence of pneumonia in low-and-middle-income countries (LMICs) despite rapid recent improvements in general living conditions, improved nutrition and better vaccines [4]. In children with pneumonia, oxygen saturation rates lower than 90–92% have been associated with an increased risk of death; therefore, hypoxia is an important prognostic indicator in paediatric pneumonia [5].

Noninvasive ventilation (NIV) is crucial in the treatment of patients with moderate to severe symptoms of respiratory distress and has been shown to reduce the need for invasive ventilation and intensive care unit (ICU) admissions [6]. Continuous positive airway pressure (CPAP) is a commonly used form of NIV that works by applying a positive pressure to the airway opening which has been shown to mitigate the reduction in functional residual capacity and to improve respiratory mechanisms and gas exchange, achieving early physiologic improvements [7]. Furthermore, there are other conditions where CPAP can be beneficial in children such as paediatric respiratory distress resulting from bronchiolitis, wheeze in the preschool child, asthma, pneumonia and nonhypercapnic acute respiratory failure [8–10]. CPAP is a suitable treatment modality for children with AHRF, with frugally engineered bubble CPAP systems used in Bangladesh, India and Malawi [11–13] shown to be effective against pneumonia and bronchiolitis, with significantly less treatment failure compared with children on standard low-flow oxygen [11].

Prioritising NIV to prevent further escalation is particularly crucial in LMICs where capacity for intensive care is extremely limited (in Uganda, for example, a country with a population of 48 million, there are approximately only 55 critical care beds [14–16]). Additionally, LMICs often have challenges including lack of medical infrastructure, lack of resources and lack of centralised oxygen supply. In some hospital environments oxygen supply may only be available from oxygen concentrators [17, 18], which typically output comparatively low pressures and offer flow rates of 5 to 10 L·min^−1^ with an oxygen concentration of around 95%. These conditions preclude the use of some NIV devices such as Venturi systems that are driven by a high-flow, high-pressure oxygen supply.

In this study, we evaluate the feasibility of using CPAP, delivered using the resource-efficient LeVe CPAP System [19], as a treatment modality among children presenting with AHRF at Mengo Hospital, Uganda. We adopted a mixed-methods approach (employing both quantitative and qualitative methods) to evaluate both the clinical outcomes and the contextual factors associated with the implementation of this innovative approach.

## Methods

**FIGURE 1:**
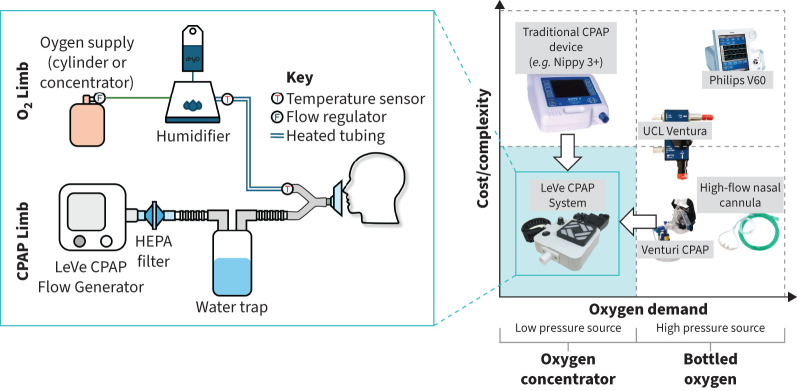
Comparison of noninvasive ventilation options for delivering continuous positive airway pressure (CPAP) (right), arranged based on their relative cost/complexity and their oxygen requirements. The LeVe CPAP System targets the lower-left “resource light” quadrant. Details of the LeVe CPAP System are shown in subfigure (callout, left). Figures adapted from Culmer *et al.* [19] and Littlejohns *et al.* [20]. HEPA: high efficiency particulate air.

The LeVe CPAP System was developed during the coronavirus disease 2019 (COVID-19) pandemic by an international and interdisciplinary team of engineers, scientists, clinicians and aid workers to meet the specific clinical requirements of LMICs. The oxygen and resource requirements of different NIV devices are summarised in figure 1: the LeVe CPAP System sits in the lower-left quadrant of this figure, having both a low complexity and a low-oxygen requirement [19]. The device consists of a frugally engineered flow generator that uses a low-voltage electric fan to generate a flow of air at clinically desirable flow rates and pressures. The level of positive end-expiratory pressure (PEEP) can be adjusted from 5 to 12 cmH_2_O and the system is inherently limited to this range to maintain safe levels of pressure. There is an independent humidified oxygen supply that can be connected to an oxygen generator or a high-pressure oxygen supply based on resource availability, and allows the oxygen supply to be controlled independently of PEEP.

The system has been developed to meet a focussed set of requirements while removing complexity, offering a resource and oxygen efficient solution that can be rapidly manufactured and deployed. The LeVe CPAP System has been previously used as a treatment modality for adults with COVID-19 [20]. The rapid deployment of the system meant that the hospital's total number of NIV devices increased from 2 to 14, with the LeVe CPAP System being recognised as a critical resource when faced with unprecedented demand for ventilatory support in critically unwell patients. Results demonstrated that the system was employed safely and successfully and was able to provide NIV support to patients who had no alternative.

This prospective study was carried out at Mengo Hospital, Uganda between November and December 2022. All the study team members (clinicians, nurses, paediatricians and biomedical engineers) were trained at the start of the recruitment period *via* an interactive presentation led by the research principal investigator and the study paediatricians. The team was trained on 1) delivery of CPAP respiratory support with the LeVe CPAP System; 2) research methods, data collection, and consistent record keeping; and 3) good clinical practices.

Approval from the Mengo Hospital Research and Ethics Committee (MHREC/106/06–2022), Mengo Hospital administration and the Uganda National Council for Science and Technology (HS2478ES) was obtained to conduct the study. Informed consent from the parents of the children was obtained before commencing treatment.

### Study subjects

Participants for inclusion to the study were children aged 1 month to 18 years admitted to the paediatric ward with AHRF, while their parents were eligible for inclusion in the qualitative study.

Inclusion criteria for patients were: children older than one month with a saturation of peripheral oxygen (*S*_pO_2__) at <88% on room air, a pulse rate above 130 bpm for all age categories, apnoea, bradypnea or cyanotic episodes (with or without bradycardia) despite supplemental oxygen, severe intercostal recession and worsening signs of respiratory distress (*e.g.* expiratory grunt, nasal flare, sternal recession), a requirement for >2 L·min^−1^ O_2_ *via* nasal prongs and inability to maintain arterial oxygen saturation (*S*_pO_2__)__ >92%, exhaustion and a high respiratory rate (RR) (>70, >60, >50 and >40 breaths·min^−1^. if aged 0–1 years, 1–2 years, 2–5 years and ≥6 years, respectively).

Patients were ineligible if parents were unable to give informed consent, the patient had a failing level of consciousness and Glasgow Coma Scale (GCS) <8, was convulsing or had severe oral ulceration, facial trauma and burns or severe deformities, or upper airway abnormalities that make CPAP ineffective or potentially dangerous (*e.g.* choanal atresia, cleft palate or trachea-oesophageal fistula). The current ceiling of care for patients in this LMIC setting was low-flow oxygen. Intubation and invasive mechanical ventilation on paediatric intensive care was not a treatment option available.

### Study design

The objective of this study was to assess the efficacy of using the resource light LeVe CPAP System for treating paediatric patients with AHRF.

The team of healthcare professionals trained to deliver the study were able to complete all aspects of the study as planned. Additionally, clinical supervisors reported improved quality of care from the trained research nurses. A total of 10 LeVe CPAP Systems were used in the study. Medical oxygen to enrich the air supply was available throughout the study and did not affect clinical decision-making (*e.g.* seeking to conserve limited supplies). Similarly, all other resources required to deliver the CPAP intervention were available, including a range of mask sizes to comfortably fit patients of a range of ages.

### Methods

Each child presenting to the emergency department with severe respiratory distress was assessed and diagnosed by the paediatrician. For those who met the study inclusion criteria, the paediatrician and study nurse informed the parents about the option of treating with the LeVe CPAP System. Parents were asked to provide written informed consent to have their children placed on the device. Correct pressure outputs of the LeVe CPAP System were confirmed by a biomedical engineer before commencing treatment.

The quantitative dataset consisted of anonymised individual-level quantitative information collected by the admitting medical officer from eligible children. The data were de-identified by the clinical team. The variables collected included the patient's demographics (age, sex and weight), date of first symptoms, date of hospital admission, date of requirement for CPAP, indication for CPAP and presence of comorbidities (such as, but not limited to, cardiac disease, congenital malformations, immunosuppression, respiratory disease such as asthma, sickle cell and HIV). Records of *S*_pO_2__, heart rate (HR), RR, PEEP, fraction of inspired oxygen, GCS, oxygen flow rate and dyspnoea (reported by trained nursing staff using a 1–10 scale, with 10 being the most severe distress [21, 22]) were taken every hour for the full duration of the treatment.

The qualitative dataset consisted of in-depth interviews using a semi-structured questionnaire recorded by the principal investigator from parents of children with AHRF. The notes included parents’ views and experiences about taking part in the study, feelings upon being informed about the need to put their child on CPAP, how they felt after seeing their child respond to treatment, what their opinion was regarding the use of the LeVe CPAP System for children with AHRF, and whether they would consent their child to future treatment with the LeVe CPAP System if it was required.

### Analysis

Key clinical measures (*S*_pO_2__, HR, RR and dyspnoea) were compared between key time points: 0 h (baseline), 6 h and 12 h to assess changes in patient condition. A further comparison was made at 72 h for patients who underwent extended treatment to assess longer-term impact of the LeVe CPAP System. The Wilcoxon signed-rank test was used to estimate the median difference between samples for each time period. Statistical analysis was conducted using R (v.4.0.4, R Core Team).

A thematic analysis was conducted identify key themes regarding parents’ experiences and perceptions of the LeVe CPAP System. The data were hand coded and datasets were analysed concurrently and independently before the results were combined for the interpretation.

## Results

A total of 42 paediatric patients were recruited onto the study ranging in age from 1 month to 17 years with 27 males and 15 females as summarised in table 1. The most common reasons for respiratory failure were pneumonia and bronchiolitis. From the total of 42 participants, 39 patients survived and were subsequently discharged, while 3 of 42 (7%) patients died. The demographics of these three patients are outlined in table 2.

**TABLE 1:** Summary of participants included in the study, outlining participant age and reason for respiratory failure diagnosed upon admission

		Number of patients	
		Male	Female
**Age range, years**	Infant (1–12 months)	20	8
	Child (1–5 years)	6	6
	Older child (5–12 years)	1	0
	Teenager (>12 years)	0	1
**Reason for respiratory failure**	Pneumonia	16	7
	Bronchiolitis	9	5
	Acute asthma exacerbation	3	2
	Other	4	3

Note that some patients presented with multiple reasons for respiratory failure, so are counted under multiple categories

**TABLE 2:** Admission details of the three patients who died during the study. All patients died within 24 h of admission

Patient	*S*_pO_2__	Heart rate	Respiratory rate	Diagnosis
**1**	83%	197	106	Severe pneumonia/bronchiolitis
**2**	80%	186	86	Severe pneumonia/bronchiolitis
**3**	89%	101	56	Acute asthma/adenoiditis

*S*_pO_2__:__ peripheral oxygen saturation.

**FIGURE 2:**
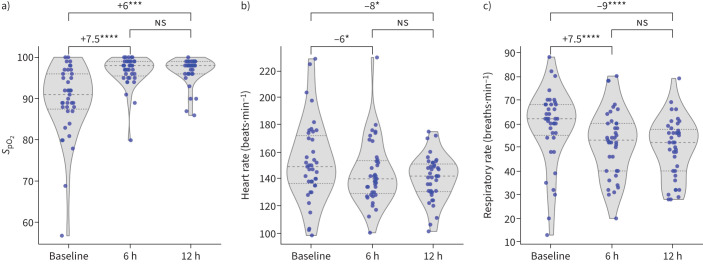
Violin plot summarising key clinical measures for patients receiving treatment with the LeVe Continuous Positive Airway Pressure System showing a) oxygen saturation, b) heart rate and c) respiratory rate at the start of the treatment (baseline), after 6 h of treatment and after 12 h of treatment. Brackets indicate the median difference between time points for each measure and the corresponding statistical significance, calculated with the Wilcoxon signed-rank test. ns: not significant; *: p≤0.05. **: p≤0.01. ***: p≤0.001. ****: p≤0.0001.

For the 39 patients that survived, there was a median length of stay of 3 days (interquartile range (IQR) 2–5, range 1–12) and a median length of treatment with the LeVe CPAP System of 3 days (IQR 1–4, range 1–8). The first 12 h of treatment saw a significant improvement of all clinical measures, as summarised in figure 2 and table 3. Values of *S*_pO_2__, HR and RR at baseline, after 6 h, and after 12 h of treatment are shown in figure 2, with the corresponding median difference in each measure calculated with the Wilcoxon signed-rank test. Results show a rapid improvement in these measures in the first 6 h of treatment, with no significant change between 6 and 12 h. For the patients that had had an extended treatment (>72 h) on the LeVe CPAP System (n=18), no adverse effects from continual use were observed, with no significant change in *S*_pO_2__, a median HR decrease of 18 bpm (95% confidence interval (CI) −8 to −29; p=0.006), and a median RR decrease of 12 (95% CI −7 to −18; p=0.003). Further plots showing the nurse-reported dyspnoea are shown in the supplementary materials.

**TABLE 3:** Summary of the clinical measures during treatment with the LeVe Continuous Positive Airway Pressure System before starting treatment (baseline), after 6 h of treatment and after 12 h of treatment

	0 h (baseline)			6 h			12 h		
	Median	IQR	Range	Median	IQR	Range	Median	IQR	Range
***S*_pO_2__**	89	87–96	57–100	97.5	92–99	80–100	98	96–99	48–100
**HR**	150	136–175	98–229	141	130–156	100–230	143	131–152	101–198
**RR**	62	56–68	13–106	54	42–61	20–80	52	40–58	28–79
**Dyspnoea**	8	6–9	4–10	7	5–8	3–10	6	5–8	3–10

IQR: interquartile range; *S*_pO_2__:__ peripheral oxygen saturation; HR: heart rate; RR: respiratory rate.

A total of five parents were interviewed to gather their experiences of the LeVe CPAP System. The children of all five parents made a full recovery and were successfully discharged from the hospital after treatment with the LeVe CPAP System. A summary of the themes is shown in table 4. None of the parents was aware of CPAP before the study, and several expressed feelings of apprehension towards the use of the device. There was a general feeling that the use of CPAP indicated a critical condition for their child. Three of the five parents that were interviewed had taken their child to hospital previously for the same condition, and among these there was a preference for the more familiar treatment of a nasal cannula. In all cases, apprehension was alleviated once the benefits of CPAP were explained by the healthcare professionals.

**TABLE 4:** Summary of the findings from the interviews conducted with five parents of the children undergoing treatment

	Before treatment	During treatment	After treatment
**+**	Apprehension alleviated once benefits explained by staff	Calmness after initial hours Relief seeing child improve Grateful for frequent checks Return to normal behaviour and feeding	Acceptance of treatment Recognition of value of CPAP Advocating CPAP to other parents (peer support)
**−**	Apprehension about new device Lack of familiarity with CPAP – preference for nasal cannula Fear that use of CPAP indicates critical state	Difficulty feeding Some discomfort around mask	

CPAP: continuous positive airway pressure. Experiences are categorised as being positive (+) or negative (−) and whether they were before, during or after the treatment with the LeVe CPAP System.

During treatment, parents had concerns around disruption to their physical interaction with their child, and multiple parents reported anxiety around disruption to feeding of their child due to the mask. Furthermore, there were reports in the initial hours of treatment of discomfort. However, as treatment with the LeVe CPAP System continued, the children's conditions improved and they were calmer; parents reported feeling relieved. Every interviewee was grateful of the frequent checks conducted on their child as part of the study.

After treatment, general perceptions of the LeVe CPAP System were positive, as the treatment had been successful. Parents recognised the value that CPAP can bring to their child, and all interviewees stated that they would consent for their child to undergo treatment in the future if required.

## Discussion

This feasibility study set out to demonstrate that the LeVe CPAP System can provide an effective intervention for children in LMIC settings presenting with AHRF. Results were gathered for 42 paediatric patients presenting to the emergency department with AHRF which met the inclusion criteria. It should be acknowledged that many of the children commenced on the LeVe CPAP System were extremely unwell, with this treatment typically offered to children who had failed conventional therapy with the real possibility of impending respiratory arrest. This can be seen by the baseline metrics reported in table 3 and figure 2, and highlights the differences in treatment pathways between LMICs and high-income countries, where many of these patients would have been admitted to an ICU. In LMICs NIV treatment such as that offered by the LeVe CPAP System is often the only option. Furthermore, in LMICs there is often a greater delay when seeking healthcare for conditions such as childhood pneumonia due to potential treatment costs, lower incomes, and challenges travelling extended distances to access appropriate healthcare facilities [23].

Of the 42 children included in this study, 3 of 42 patients (7% of total) sadly passed away within 3–6 h of admission to hospital despite the intervention. Their deaths were not related to device malfunction or failure, and the critical state that these patients were in when they began treatment should be noted (table 2). Additionally, the national referral hospital, where these patients would have received more advanced care, lacks CPAP or invasive mechanical ventilator support specifically for children. This once again highlights the health inequalities and the different use of cases of CPAP devices between high and low-income countries. The concern in high-resource settings that high-flow oxygen or CPAP therapies can act as a delay to intubation and invasive mechanical ventilation in some children is not valid in this study population. The 39 patients who survived treatment showed a consistent and significant improvement in key physiological parameters in the first 12 h of treatment: an increase in *S*_pO_2__, a decrease in HR and stabilisation in RR. Furthermore, it was demonstrated that longer-term use of the LeVe CPAP System (>72 h) did not lead to any adverse effects in these metrics. The median length of stay was 3 days, and all surviving patients were discharged following treatment.

The interviews conducted with the patients’ parents revealed similar themes across all five interviewees. Before treatment, there was a general feeling of apprehension, in part due to the lack of familiarity with the use of CPAP. If CPAP treatment becomes more accessible and widespread, these concerns may be alleviated. In fact, interviewees already reported that they were advocating the LeVe CPAP System to other parents whose children were undergoing treatment to alleviate their concerns. Many of the parents’ other concerns during treatment revolved around issues with the masks, namely issues with feeding their child and discomfort due to the pressure of the mask and the drying out of mucous membranes. These issues have been reported elsewhere [24–26] and are particularly prevalent in paediatric patients. These results highlight the importance of having the correct ancillary equipment and consumables (*e.g.* masks, tubing, filters and humidifiers) to support safe and sustainable delivery of CPAP. It is important to consider the supply chain, to ensure that these components can be reliably procured within an LMIC setting. Where possible, adoption of reusable components that can be cleaned, sterilised and reused on site can help mitigate this risk and reduce long-term costs in comparison with single-use items.

By the end of the treatment, all interviewees spoke positively about the LeVe CPAP System. It should be noted that all interviewees were parents of patients who survived treatment. Parents whose child had been to hospital previously reported that they had seen faster improvements with the LeVe CPAP System than with previously used treatments, but these remain anecdotal reports and should be substantiated with data from comparative studies. Several interviewees also stated that they were grateful of the more frequent checks performed as part of the study – this highlights the importance of appropriate staffing levels and professional training, in addition to equipment availability. It is important that as use of the LeVe CPAP System increases, the number of staff trained to administer treatment with the device increases proportionally. This has been highlighted in works where CPAP has been evaluated in LMIC settings, with one study stating that a 1:4 nurse to patient ratio is required for the therapy to be safe and effective [27].

The LeVe CPAP System has been designed with frugal engineering concepts to create a NIV device that is both cost-effective and does not rely on a high-flow oxygen supply. The availability of the LeVe CPAP System provided a safe and effective treatment option for paediatric patients suffering from AHRF who may otherwise have had no alternative option. Further work should assess the efficacy of the LeVe CPAP System by comparing it directly to other treatment modalities, although we appreciate that this is challenging in LMIC settings due to the limited resources.

The LeVe CPAP System is the result of a truly collaborative piece of multidisciplinary research co-development between clinicians, engineers and academics from Leeds (UK) and Kampala (Uganda) to address the critical clinical need for resource-effective NIV in LMICs. This study builds on that work, demonstrating that the system has virtue in paediatric treatment pathways. The outcomes support the need for further translation to support the potential for wider adoption of the LeVe CPAP System in treating paediatric patients with AHRF, offering life-saving treatment options for critically unwell patients in LMICs.

### Study limitation

The main limitation of this study is the small sample size for both the qualitative and quantitative components. The qualitative data reflect the experiences and reports of only five families or patients, which are largely anecdotal and may not be transferable to other contexts or populations. Similarly, the quantitative data, comprising only 42 participants, may limit the generalisability of the findings. These constraints should be considered when interpreting the study's results.

## Supplementary material

**Please note:** supplementary material is not edited by the Editorial Office, and is uploaded as it has been supplied by the author. Supplementary material 00673-2024.SUPPLEMENT
