# Supplementary material for: Feasibility of using continuous positive airway pressure via the LeVe CPAP System among children with acute hypoxaemic respiratory failure at Mengo Hospital, Kampala, Uganda: a mixed-methods study
Source: ERJ Open Res. 2025 Jun 30;11(3):00673-2024. doi: 10.1183/23120541.00673-2024 (PMC12208565; doi:10.1183/23120541.00673-2024)
Supplement: Supplementary file 1 [file 00673-2024.SUPPLEMENT.pdf]

Feasibility of using Continuous Positive Airway Pressure via the 'LeVe CPAP System' among Children with Acute Hypoxaemic Respiratory Failure at Mengo Hospital Kampala Uganda: A Mixed Methods study

Namulema, Edith[1]; **Davis Birch**, William[3]; **Nakiriba Mayega**, Rhoda[1]; **Namugga**, Barbara[1]; **Musasizi**, Racheal[1]; **Tumwesigye**, Ambrose[1]; **Littlejohns**, Anna[2]; **Please**, Helen[2]; **Sharma**, Vishal[5]; **Cunningham**, Alice[5]; **Waters**, Ian[3]; **Brettle**, David[2]; **Parmar**, Jiten D[2]; **Miller**, Roy[4]; **Beacon**, Tim[4]; **Murdoch**, Stuart[2]; **Culmer**, Peter[3]; **Kapur**, Nikil[3]; **Winton**, Mark[2]; **Lawton**, Tom[5]

Corresponding author: Dr Edith Namulema, edith.namulema@mengohospital.org

Take home message:

The LeVe CPAP System provides effective respiratory support for paediatric patients with hypoxaemic respiratory failure in low-resource contexts, improving patient outcomes and minimising the need for further treatment escalation.

Supplemental information

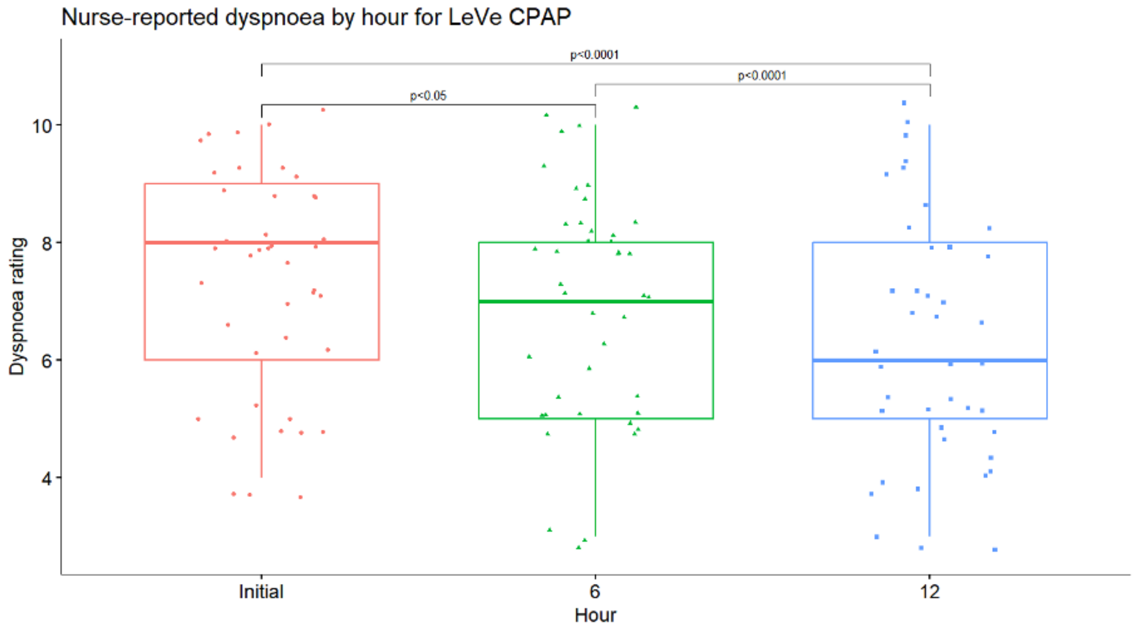

Figure S1: Dyspnoea for patients using the LeVe CPAP System. Dyspnoea is reported on a 10-point scale, with 10 being the most severe dyspnoea [1, 2]. Data is shown for initial conditions (on admission), and after 6 and 12 hours of treatment on the LeVe CPAP System.

References

1. Mahler DA and Horowitz MB, *Perception of breathlessness during exercise in patients with respiratory disease.* Medicine & Science in Sports & Exercise, 1994. **26**(9): p. 1078-1081.
2. Crisafulli E and Clini EM, *Measures of dyspnea in pulmonary rehabilitation.* Multidiscip Respir Med, 2010. **5**(3): p. 202-10.
